# Nonsterile l-Lysine Fermentation Using Engineered Phosphite-Grown *Corynebacterium glutamicum*

**DOI:** 10.1021/acsomega.1c00226

**Published:** 2021-04-07

**Authors:** Ming Lei, Xiwei Peng, Wenjun Sun, Di Zhang, Zhenyu Wang, Zhengjiao Yang, Chong Zhang, Bin Yu, Huanqing Niu, Hanjie Ying, Pingkai Ouyang, Dong Liu, Yong Chen

**Affiliations:** † National Engineering Research Center for Biotechnology, College of Biotechnology and Pharmaceutical Engineering, Nanjing Tech University, Nanjing 211816, China; ‡ State Key Laboratory of Materials-Oriented Chemical Engineering, College of Biotechnology and Pharmaceutical Engineering, Nanjing Tech University, Nanjing 211816, China; § School of Chemical Engineering and Energy, Zhengzhou University, Zhengzhou 450001, China

## Abstract

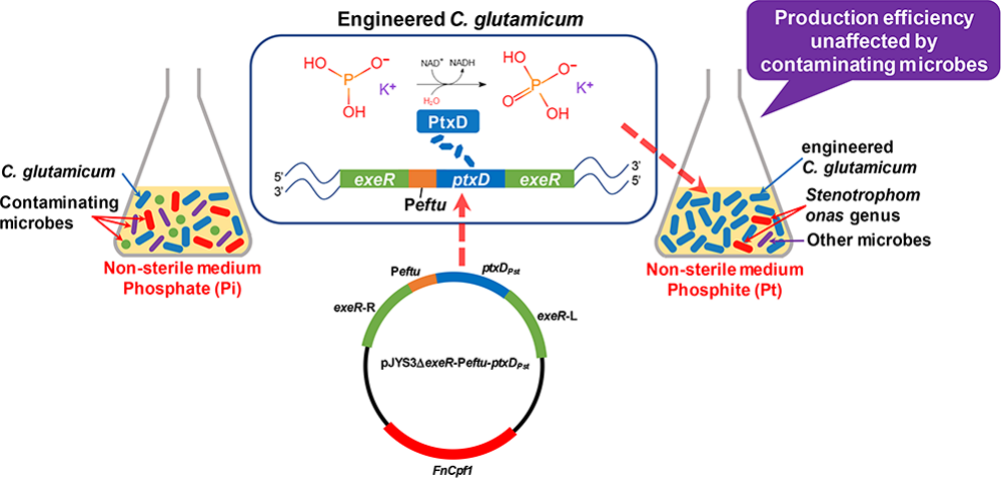

Fermentation using *Corynebacterium glutamicum* is an important method for the industrial production of amino acids. However, conventional fermentation processes using *C. glutamicum* are susceptible to microbial contamination and therefore require equipment sterilization or antibiotic dosing. To establish a more robust fermentation process, l-lysine-producing *C. glutamicum* was engineered to efficiently utilize xenobiotic phosphite (Pt) by optimizing the expression of Pt dehydrogenase in the *exeR* genome locus. This ability provided *C. glutamicum* with a competitive advantage over common contaminating microbes when grown on media containing Pt as a phosphorus source instead of phosphate. As a result, the engineered strain could produce 41.00 g/L l-lysine under nonsterile conditions during batch fermentation for 60 h, whereas the original strain required 72 h to produce 40.78 g/L l-lysine under sterile conditions. Therefore, the recombinant strain can efficiently produce l-lysine under nonsterilized conditions with unaffected production efficiency. Although this anticontamination strategy has been previously reported for other species, this is the first time it has been demonstrated in *C. glutamicum*; these findings should aid in the further development of cost-efficient amino acid fermentation processes.

## 1 Introduction

*Corynebacterium glutamicum* is used for the industrial production of various amino acids and other chemicals.^1−4^ It is the most important strain used for the production of l-glutamate and l-lysine, which have the largest commercial demand among various amino acids.^5^ The global market for l-lysine and l-glutamate is estimated to be more than 2.5 and 3 million tons per year, respectively.^6^ However, amino acid fermentation by *C. glutamicum* is susceptible to microbial contamination, which usually leads to fermentation failure and huge economic losses.^7^ Methods to prevent microbial contamination are typically process sterilization and the addition of antibiotics.^8^ However, the abuse of antibiotics will accelerate the emergence of resistant strains.^9,10^ Process sterilization results in high-energy consumption and high cost, and steam-based sterilization of culture media can also cause the Maillard reaction to occur.^11^ The Maillard reaction is the reaction of sugars and amino acids in the fermentation media, which often leads to the loss of nutrients and thus reduces the yield of the product.^12^ Therefore, there is an urgent need to develop strategies to reduce the risk of microbial contamination.^13^

Modifying the production strain is one way to address the problem of microbial contamination in the industrial fermentation process. If a production strain has a competitive advantage over contaminating strains during fermentation, sterilization may be omitted thereby simplifying the production operation, reducing the production costs, and the increasing product yield. Phosphite (Pt) dehydrogenase (PtxD) is an NAD^+^-dependent enzyme that can convert Pt to phosphate (Pi) with concomitant reduction of NAD^+^ to NADH.^14^ PtxD is also of interest in the field of coenzyme regeneration.^15−17^ If PtxD was added to a bacterial strain, the engineered strain could grow in a fermentation medium supplemented with only Pt as the phosphorus source thereby gaining an advantage when competing with contaminating microbes. Thus, the engineered cells would become the dominant population in a contaminated system, and batch fermentation could be carried out in a nonsterile medium.

Strains have been engineered in recent years to acquire the capability of utilizing Pt as a phosphorus source.^18,19^ Pt is classified as an organic fertilizer and has been approved for use on food crops because it is nontoxic to humans and animals.^20^ In 2014, the Pt dehydrogenase gene *ptxD* was introduced into *Schizosaccharomyces pombe* and *Saccharomyces cerevisiae* to combat microbial contamination during the fermentation process.^21^ In 2018, *ptxD* was introduced into *cyanobacteria*,^22^ and in 2020, formamidase (*fmdA*) and *ptxD* genes were introduced into *Bacillus subtilis* for fermentation in nonsterile systems.^23^ However, there have been no reports of *C. glutamicum*, one of the most important amino acid production strains in industry, being modified in this fashion. Whether Pt assimilation is effective in *C. glutamicum* fermentation processes to combat microbial contamination remains to be investigated. Using Pt as a phosphorus source for *C. glutamicum* may lay the foundation for the industrial production of amino acids under nonsterile conditions. In this study, an optimal *ptxD* gene was inserted into the genome of *C. glutamicum* at a particular gene site (the *exeR* gene site) using the CRISPR-cpf1 technology for fermentation in a nonsterile medium.

## 2 Results and Discussion

### 2.1 Plasmid-Based Expression of *ptxD* Genes in *C. glutamicum*

**Figure 1:**
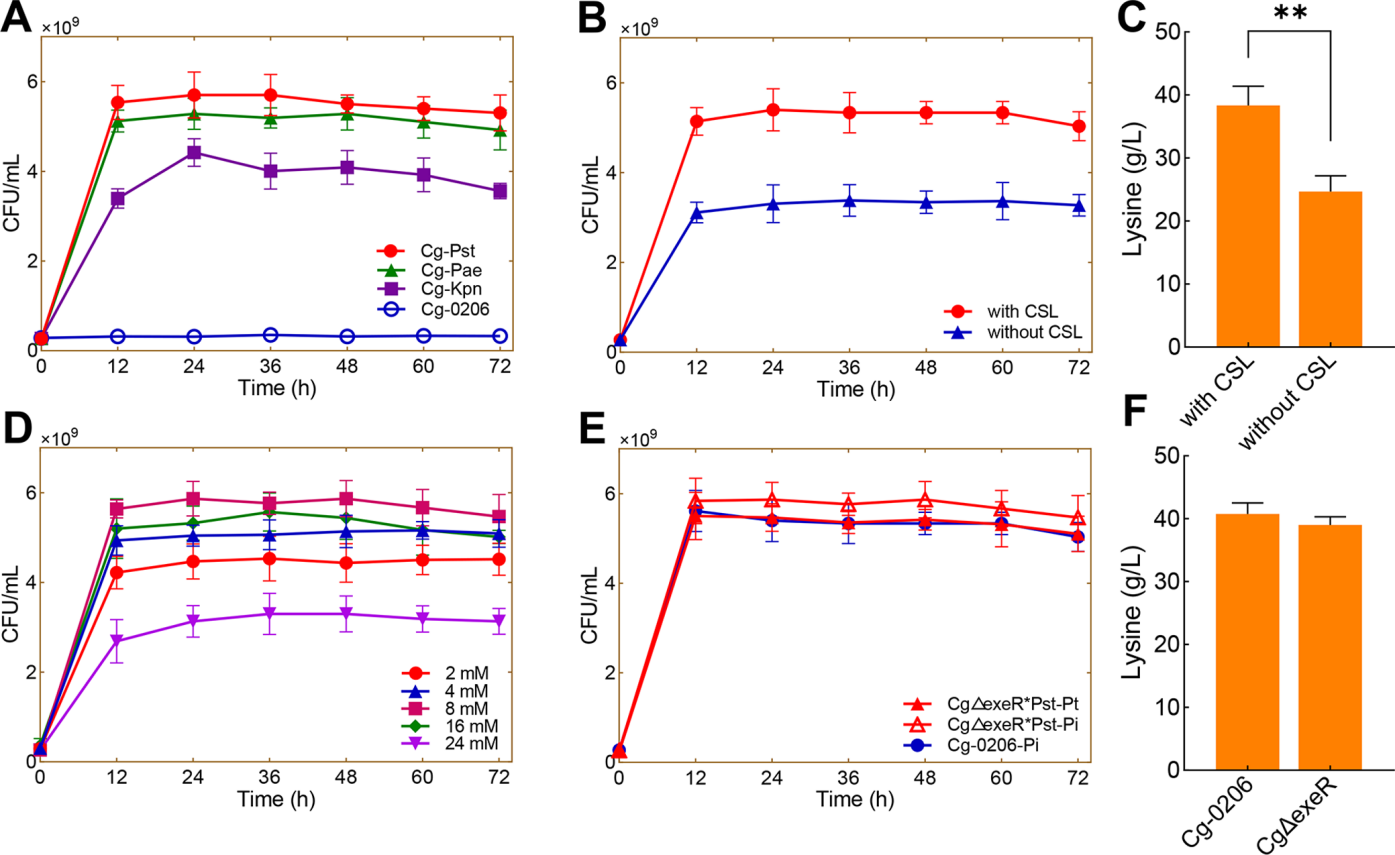
Construction of engineered *C. glutamicum*. (A) Growth curves of Cg-Pst, Cg-Pae, Cg-Kpn, and Cg-0206 in the Pt medium. (B) Growth curves of Cg-0206 in the Pi medium (with CSL and without CSL). (C) Lysine yield of Cg-0206 in the Pi medium (with and without CSL). (D) Effect of Pt concentration on the growth of Cg-Pst in the Pt medium. (E) Growth curves of Cg-0206 and CgΔexeR*Pst in the Pt or Pi medium. (F) Lysine yield of Cg-0206 and CgΔexeR in the Pi medium. Error bars are given showing standard deviations for *n* = 3. ***p < 0.001, **p < 0.01, *p < 0.05 using Student’s *t*-test.

Three different *ptxD* genes from *Pseudomonas stutzeri*, *Pseudomonas aeruginosa*, and *Klebsiella pneumoniae* were individually introduced into Cg-0206, and the resultant strains were named Cg-Pst, Cg-Pae, and Cg-Kpn, respectively. These engineered strains were cultured on a Pt medium to test their Pt utilization capability. Compared with a conventional fermentation medium, the Pt medium used Pt as the sole phosphorus source and omitted corn steep liquor (CSL), which might contain naturally occurring Pi from the fermentation medium. As shown in Figure 1A, all three engineered strains could grow on the Pt medium, with Cg-Pst demonstrating the best growth performance. In contrast, the original Cg-0206 strain barely grew on the Pt medium. Hence, the *ptxD* gene from *P. stutzeri* was the best for *C. glutamicum* growth. A previous study had similarly found that *ptxD* from *P. stutzeri* was better than other genes that were tested in *B. subtilis*.^23^

Because the original Cg-0206 strain could not grow on the Pt medium, a conventional Pi fermentation medium (Pi medium) but without CSL was used to determine its growth. As shown in Figure 1B, the growth of Cg-0206 was reduced by 35.0% (from 5.03 to 3.27 cfu/mL) and l-lysine production was reduced by 35.6% (from 38.33 to 24.67 g/L after 72 h, Figure 1C) by omitting CSL. Although the growth of Cg-0206 was significantly affected by CSL omission, the growth of Cg-Pst in the Pi medium without CSL was not affected. This indicated that CSL probably did contain naturally occurring Pi, and thus, the removal of CSL would be critical for the engineered strain to outcompete other strains. The removal of CSL would also help to further reduce feedstock cost.

The effect of Pt concentration on cell growth was determined by culturing Cg-Pst with 2–24 mM KH_2_PO_3_. The best growth performance was obtained at a concentration of 8 mM (Figure 1D). This molar concentration is the same as that of Pi in the conventional Pi medium. Therefore, the amount of phosphorus required for fermentation did not change for the engineered strain.

### 2.2 Genome-Integrated Expression of the *ptxD*_*pst*_ Gene in *C. glutamicum*

To further enhance the genetic stability of the engineered strain, the *Peftu*-*ptxD*_*Pst*_ expression cassette was integrated into the genome of Cg-0206 using the CRISPR technique. The *exeR* locus (NCgl2503) of the Cg-0206 genome was selected as the insertion site for *ptxD*_*Pst*_. A recent study showed that inactivation of *exeR*, which codes for an extracellular nuclease, in an l-proline producing *C. glutamicum* effectively increased extracellular DNA (eDNA) abundance, thereby facilitating biofilm formation and greatly increasing the efficiency of biofilm-based continuous (repeated batch) fermentation.^24^ Therefore, *exeR* was selected as the insertion site because it might help establish a long-term continuous fermentation process under the desired nonsterile conditions. Deletion of *exeR* in Cg-0206, performed here for the first time, generated a strain named CgΔexeR. Deletion of *exeR* did not affect l-lysine production (from 40.77 to 39.00 g/L) during conventional batch fermentation (Figure 1F), demonstrating the feasibility of *exeR* as a genome insertion site. Subsequently, the *ptxD*_*pst*_ gene was integrated into the genome at the *exeR* site in Cg-0206, generating a *ptxD*-expressing, *exeR*-disrupted strain named CgΔexeR*Pst. To verify whether genetic modification affected cell fitness, we cultured the original Cg-0206 strain and the recombinant CgΔexeR*Pst strain in Pi and Pt media, respectively. We found that the genetic modification of Cg-0206 did not affect cell fitness (Figure 1E). In fact, the recombinant strain showed the best growth performance in the Pt fermentation medium (Pt medium).

### 2.3 Competitive Advantage of CgΔexeR*Pst over Contaminating Microbes on the Pt Medium

**Figure 2:**
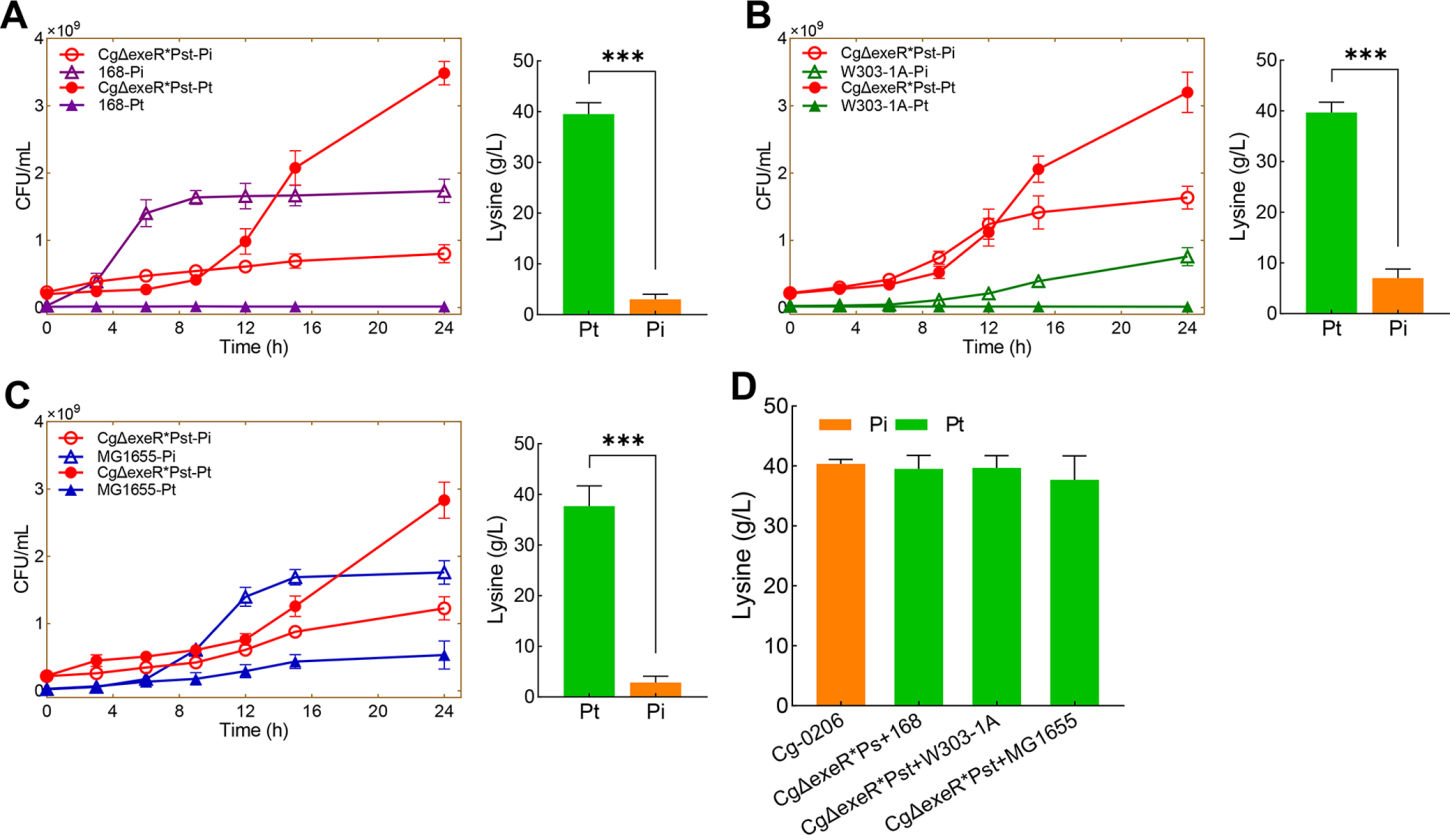
Competitive experiments with different strains in the Pi medium or Pt medium. (A) Coculturing competition experiments of CgΔexeR*Pst and *B. subtilis* 168 in the Pi medium and the Pt medium. (B) Coculturing competition experiments of CgΔexeR*Pst and *S. cerevisiae* W303-1A in the Pi medium and the Pt medium. (C) Coculturing competition experiments of CgΔexeR*Pst and *E. coli* MG1655 in the Pi medium and the Pt medium. (D) Lysine yield of Cg-0206 and the coculture system in the Pi medium or Pt medium, respectively. Error bars are given showing standard deviations for *n* = 3. ***p < 0.001, **p < 0.01, *p < 0.05 using the Student’s *t*-test.

One of the most common contaminating strains in *C. glutamicum* fermentation processes is *B. subtilis*. To evaluate the competitive advantage of CgΔexeR*Pst, *B. subtilis* 168, *Escherichia coli* MG1655, and *S. cerevisiae* W303-1A were used as representative contaminating strains and were individually cocultured with CgΔexeR*Pst at an initial inoculum ratio of 1:9 (v/v) (see Method Section 3.5 for details). The results showed that both *B. subtilis* and *S. cerevisiae* grew when cultured on a conventional Pi medium. In fact, *B. subtilis* grew faster than CgΔexeR*Pst. However, when a Pt medium was used, neither *B. subtilis* nor *S. cerevisiae* grew (Figure 2). In contrast, CgΔexeR*Pst grew on the Pt medium to a high cell density of 3–4 × 10^9^ cfu/mL. In the fermentation process using the cocultivation of CgΔexeR*Pst and contaminated strains, the lysine production efficiency of CgΔexeR*Pst could be restored by replacing the Pi medium with a Pt medium. Lysine production under a cocultured system with *B. subtilis* or *S. cerevisiae* was restored from 3.00 to 39.5 and from 7.00 to 39.67 g/L, respectively (Figure 2A,B).

Unlike *B. subtilis* and *S. cerevisiae*, *E. coli* harbors a protein with phosphite dehydrogenase activity.^25^ Thus, *E. coli* can grow on both Pi and Pt fermentation media. On the Pi medium, the cell density of *E. coli* was slightly higher than that of CgΔexeR*Pst (Figure 2C). Nevertheless, on the Pt medium, CgΔexeR*Pst outcompeted *E. coli,* and a cell density five times higher than that of *E. coli* (Figure 2C) was obtained. Therefore, although *E. coli* was not completely inhibited on the Pt medium, lysine production still reached 37.67 g/L (Figure 2C).

Overall, growth competition experiments demonstrated that the selective pressure generated by Pt endowed CgΔexeR*Pst with the capability to dominate the population in a multispecies system. Furthermore, when CgΔexeR*Pst was cultured in a Pt medium, l-lysine production was comparable to that in the conventional Pi medium (Figure 2D). These results suggest that CgΔexeR*Pst is suitable for l-lysine fermentation under nonsterile conditions.

### 2.4 Nonsterile Fermentation and Microbial Population Analysis

**Figure 3:**
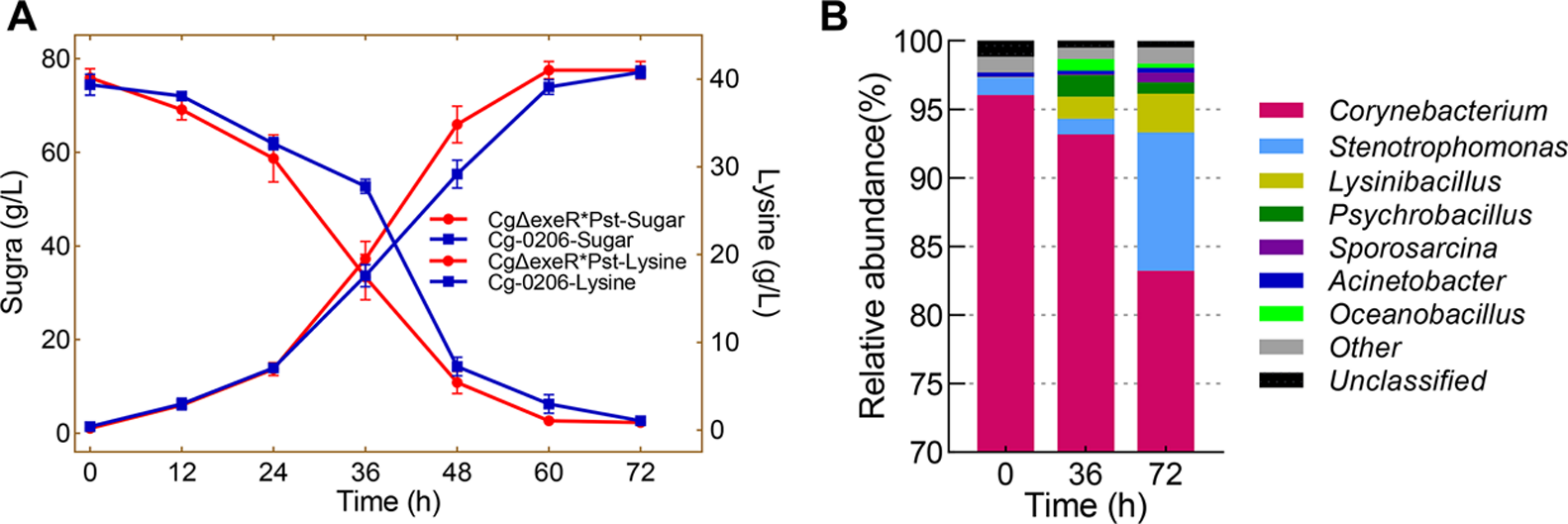
Nonsterile fermentation and microbial population analysis. (A) l-Lysine production and sugar consumption in batch fermentation by CgΔexeR*Pst in a nonsterile Pt medium. (B) Microbial population analysis of the nonsterile fermentation broth based on the 16S rRNA sequences by high-throughput sequencing. Error bars are given showing standard deviations for *n* = 3.

The engineered strain CgΔexeR*Pst was used under nonsterile conditions for l-lysine fermentation. The fermentation medium and culture flasks were not sterilized, and operations such as inoculation and sampling were performed under nonsterile conditions. The results showed that when CgΔexeR*Pst was cultured in a nonsterile Pt medium, l-lysine production reached 41.00 g/L (Figure 3A). Compared with the original Cg-0206 strain cultured under sterile conditions, the sugar consumption speed of CgΔexeR*Pst was faster, and the fermentation period was shortened from 72 to 60 h (Figure 3A). Therefore, using CgΔexeR*Pst to produce l-lysine under nonsterile conditions could simplify the fermentation process, shorten the fermentation cycle, and ultimately reduce the production costs.

Because the fermentation medium, shake flasks, sampling, and other operations were not performed in a sterile environment, it was suspected that some environmental microbes might grow in the fermentation medium. Therefore, the microbial populations in the Pt fermentation broth during nonsterile fermentation were analyzed. As shown in Figure 3B, *Corynebacterium* remained the dominant population throughout the fermentation process. It comprised 93.18% of the population at 36 h and 83.22% at the end of fermentation (72 h), explaining the unaffected fermentation performance. The contaminating microbes were found to belong to the genera *Stenotrophomonas*, *Acinetobacter*, *Lysinibacillus*, *Psychrobacillus*, *Sporosarcina*, and *Oceanobacillus*. The most notable contaminating microbes at the end of fermentation were members of the *Stenotrophomonas* genus (10.11% of the population). Bioinformatics analysis suggested that *Stenotrophomonas* had a PtxD-like dehydrogenase, while other contaminating genera had no apparent PtxD, with the exception of *Acinetobacter*. This indicated that environmental microbes with native Pt utilization ability were most likely to be contaminating strains. Other strains could also coexist during fermentation in a much smaller proportion, probably by relying on metabolites from other strains to grow.

### 2.5 Trial of Nonsterile Continuous Fermentation

Because CgΔexeR*Pst performed well in batch fermentation under nonsterile conditions, the possibility of using it for long-term continuous fermentation under nonsterile conditions was evaluated. To do this, fermentation was carried out in a repeated batch fermentation mode using either free cells or biofilm-immobilized cells^24^ under nonsterile conditions. In the nonsterile repeated batch fermentation, l-lysine production gradually decreased over the fermentation batches. The production of l-lysine by CgΔexeR*Pst free cells in the third batch of fermentation was only 66.7% of that in the first batch (from 41.00 to 27.33 g/L, Figure S1). The performance of biofilm-immobilized cells was even worse, with l-lysine production in the third batch being only 35.3% of that in the first batch (from 37.67 to 13.30 g/L, Figure S2).

## 3 Materials and Methods

### 3.1 Strains, Media, and Growth Conditions

Strains and plasmids used in this work are listed in Table 1. *C. glutamicum* 0206 (Cg-0206) was an l-lysine producer derived from *C. glutamicum* CICC 21763 through mutagenesis. All plasmids were introduced by chemical transformation into competent cells of *E. coli* DH5α that was grown in Luria–Bertani broth (LB) at 37 °C. *C. glutamicum* strains were routinely cultured in the LBG medium (LB supplemented with 10 g/L glucose) at 30 °C unless otherwise indicated. The amount of antibiotic added for recombinant strains was as follows: kanamycin 50 mg/L, chloramphenicol 50 mg/L for *E. coli* and kanamycin 25 mg/L, chloramphenicol 25 mg/L for *C. glutamicum*.

**Table 1:** Strains and Plasmids Used in This Study

strains or plasmids	relevant characteristics	sources
Strains		
*C. glutamicum* CICC 21763	l-lysine-producing strain	CICC
*C. glutamicum* 0206	derived from CICC 21763 through mutagenesis	laboratory stock
*E. coli* DH5α	plasmids holding strain	laboratory stock
*E. coli* MG1655	wild-type strain	laboratory stock
*B. subtilis* 168	wild-type strain	laboratory stock
*S. cerevisiae* W303-1A	wild-type strain	laboratory stock
CgΔexeR	*C. glutamicum* 0206 with ∼1 kb deletion of *exeR*	this study
Cg-Pst	*C. glutamicum* 0206 with pXMJ19*ptxD_*Pst*_	this study
Cg-Pae	*C. glutamicum* 0206 with pXMJ19*ptxD_*Pae*_	this study
Cg-Kpn	*C. glutamicum* 0206 with pXMJ19*ptxD_*Kpn*_	this study
CgΔexeR*Pst	*C. glutamicum* 0206 with ∼1 kb deletion of *exeR*, P*eftu*, and *ptxD*_*Pst*_ inserted at this deletion region	this study
Plasmids		
pXMJ19	<keep-together>*E. coli*–*C. glutamicum*</keep-together> shuttle vector; Cm^r^ Ptac lacIq pMB1 oriV*E. coli* pBL1 oriV*C. glutamicum*	(26)
pXMJ19*ptxD_*Pst*_	pXMJ19 containing P*eftu* and *ptxD*_*Pst*_	this study
pXMJ19*ptxD_*Pae*_	pXMJ19 containing P*eftu* and *ptxD*_*Pae*_	this study
pXMJ19*ptxD_*Kpn*_	pXMJ19 containing P*eftu* and *ptxD*_*Kpn*_	this study
pJYS3_*crtYf*	pBL1^ts^*oriVC. glutamicum* Kn^r^ pSC101 *oriVE. coli* PlacM-FnCpf1, Pj23119-crRNA targeting *crtYf*, 1 kb upstream and downstream homologous arms flanking 705-bp deletion fragment inside *crtYf*	(26)
pJYS3Δ*exeR*	pBL1^ts^*oriVC. glutamicum* Kn^r^ pSC101 *oriVE. coli* PlacM-FnCpf1, Pj23119-crRNA targeting *exeR*, 1 kb upstream and downstream homologous arms flanking 1125-bp deletion fragment inside *exeR*	this study
pJYS3Δ*exeR*-P*eftu*-*ptxD*_*Pst*_	derived from pJYS3_Δ*exeR*; P*eftu* (290 bp) and *ptxD*_*Pst*_ (∼1 kb) inserted between the 1 kb upstream and downstream homologous region flanking the 1125-bp deletion fragment inside *exeR*	this study

To prepare the seed culture for fermentation, the strain was transferred from an LBG agar plate into a 500 mL flask containing 50 mL of seed medium (25 g/L sucrose, 10 g/L tryptone, 5 g/L yeast extract, 5 g/L urea, 5 g/L KH_2_PO_4_, 12 g/L K_2_HPO_4_, 1 g/L MgSO_4_·7H_2_O, 5 g/L (NH_4_)_2_SO_4_) and incubated at 30 °C, 220 rpm for 6–8 h. Then, 10 mL of the seed culture was added into another 500 mL flask containing 50 mL fermentation medium. The Pi medium contained 100 g/L glucose monohydrate, 20 mL/L beet molasses, 20 mL/L CSL, 1.1 g/L KH_2_PO_4_, 10 mg/L thiamin, 2 mg/L biotin and 50 mg/L nicotinamide, 10 mg/L d-calcium pantothenate, 1 g/L MgSO_4_·7H_2_O, 40 g/L (NH_4_)_2_SO_4_, 0.15 g/L FeSO_4_·7H_2_O, 1 mg/L CuSO_4_·5H_2_O, 1 mg/L ZnSO_4_, 0.1 g/L MnSO_4_, and 40 g/L CaCO_3_. The Pt medium was the same as the Pi medium, except that KH_2_PO_4_ was replaced by KH_2_PO_3_ and the CSL was removed. The initial pH of the seed medium was adjusted to 7.2 using KOH. Unless indicated, all media were sterilized at 115 °C for 20 min. l-Lysine and sugar concentrations were measured using an immobilized enzyme biosensor (SBA-40E, Shandong, China).

### 3.2 Plasmid-Based Gene Expression

All primers used in this study are listed in Table 2. Three different *ptxD* genes from *P. stutzeri*,^22^*P. aeruginosa,*^27^ and *K. pneumoniae*(28) were tried. The *ptxD* genes were expressed based on the pXMJ19 plasmid under the P*eftu* promoter. The P*eftu* promoter were amplified from the pJYS3_*crtYf* plasmid using primers *Peftu-F/*Peftu-R. The *ptxD*_*Pst*_, *ptxD*_*Pae*_, and *ptxD*_*Kpn*_ gene fragments (with sequences homologous to plasmid pXMJ19) were codon-optimized and synthesized by Nanjing Tsingke Biological Technology Co., Ltd. The P*eftu* promoter and each of the *ptxD* genes were ligated into BamHI-linearized pXMJ19 plasmid, generating recombinant plasmid pXMJ19*ptxD_*Pst*_, pXMJ19*ptxD_*Pae*_, and pXMJ19*ptxD_*Kpn*_, respectively. The ligation was accomplished through homologous recombination using the ClonExpress Ultra One Step Cloning Kit (Vazyme) according to the manufacturer’s protocol.

**Table 2:** Primers Used in This Study

primer	sequence
*Peftu-F	GCCTGCAGGTCGACTCTAGAGGATCCAGATCAGTAGGCGCGTAGGG
*Peftu-R	AGCATTGTATGTCCTCCTGGACTTC
exeR-R-F	AAGTAGAACAACTGTTCACCGGGCCCACGGAATCATCTACC
exeR-R-R	GGCGTGCTGGAGTCGGTTCCGGCAGGATTA
exeR-L-F	TAATCCTGCCGGAACCGACTCCAGCACGCC
exeR-L-R	TGAGCTAGCTGTCAATCTAGAGCGTCGAATTCGGT
crRNA-exeR-F	ACGCTCTAGATTGACAGCTAGCTCA
crRNA-exeR-R	CTGAGCCTTTCGTTTTATTTAAATGTAACGCTCCAACCGTCGAGGATCTACAACAGTAGA
pJYS3-Pst-F	TAATCCTGCCGGAACAGATCAGTAGGCGCG
pJYS3-Pst-R	GGCGTGCTGGAGTCGTTAACATGCGGCTGG

### 3.3 Genome-Integrated Gene Expression

Deletion of *exeR* and genome-integrated expression of *ptxD* was performed using the pJYS3 plasmid based on the CRISPR technology previously published.^26^ To delete *exeR*, the *C. glutamicum* genome was used as a template to amplify homologous arms that were 1000 bp upstream and downstream of *exeR* using primers exeR-R-F/exeR-R-R and exeR-L-F/exeR-L-R, respectively. The original targeting sequence crRNA on pJYS3_*crtYf* was amplified and retargeted to *exeR* using primers crRNA-exeR-F/crRNA-exeR-R. Subsequently, the homologous arms and the retargeted crRNA were ligated into ApaI/SwaI-linearized pJYS3_*crtYf* using the One Step Cloning Kit mentioned above, generating a recombinant plasmid pJYS3Δ*exeR*. To integrate the *ptxD*_*pst*_ gene into the *exeR* locus of the *C. glutamicum* genome, the same procedures were followed, except that the P*eftu*-*ptxD*_*pst*_ expression cassette on pXMJ19*ptxD_*Pst*_ was amplified using primers pJYS3-Pst-F/pJYS3-Pst-R and inserted between the homologous arms, generating a recombinant plasmid pJYS3Δ*exeR*-P*eftu*-*ptxD*_*pst*_.

All plasmids were delivered into *C. glutamicum* through electroporation. Electroporation of *C. glutamicum* and curing of the pJYS3-derived plasmids were performed according to a previously published method.^26^

### 3.4 Coculture Competition Experiment

To set up the cocultures, *E. coli* MG1655, *S. cerevisiae* W303-1A, and *B. subtilis* 168 were routinely grown in LB, YPD, and LB medium for seed culture, respectively. The OD_600_ of the seed cultures as well as the CgΔexeR*Pst seed culture was all adjusted to 7.5. Then, 1 mL of each seed culture was individually mixed with 9 mL of CgΔexeR*Pst seed culture. 10 mL of mixture was used to inoculate 50 mL of Pi medium or Pt medium in 500 mL shake flasks maintained at 30 °C with 220 rpm agitations. To determine the population fraction, coculture of CgΔexeR*Pst and *E. coli* and coculture of CgΔexeR*Pst and *B. subtilis* were plated in triplicate on LBG agar medium, while the coculture of CgΔexeR*Pst and *S. cerevisiae* were plated on both LBG and YPD agar media. After incubation at 30 °C for 1–2 d, the colony forming units per mL (cfu/mL) were calculated. Colonies of the strains were distinguished by the colony morphology.

### 3.5 Batch Fermentation and Repeated Batch Fermentation

For batch fermentation, 10 mL of seed culture was grown to an OD_562_ of 8.0–9.0 and was used to inoculate 50 mL of Pt medium or Pi medium in a 500 mL flask and then incubated aerobically at 30 °C, 220 rpm for 72 h. For nonsterile fermentation, the seed culture was centrifuged to collect the cells that were used to inoculate nonsterile medium in tap water-washed nonsterile flasks, and all the operations such as inoculation were performed under nonsterile conditions.

For free-cell repeated batch fermentation, 10 mL of fermentation broth at the end of each batch fermentation was retained as seed culture and then, 50 mL of fresh nonsterile Pt medium together with 2 g of CaCO_3_ was added to initialize the next batch fermentation. For adsorption-immobilized (also called biofilm-based) repeated batch fermentation, a cotton towel was added as the cell carrier, according to a previous experimental design.^29^ Other conditions were the same as for free-cell repeated batch fermentation, except those at the end of each batch fermentation. All the fermentation broth was replaced with 60 mL of fresh Pt medium and 2 g of CaCO_3_ to initialize the next batch fermentation.

## 4 Conclusions

To our knowledge, this is the first report on nonsterile l-lysine fermentation using engineered *C. glutamicum*. The engineered strain could produce l-lysine under nonsterile conditions during batch fermentation without affecting the production efficiency. Compared with the original Cg-0206 strain cultured under sterile conditions, this nonsterile fermentation can simplify the fermentation process, shorten the fermentation cycle, and ultimately reduce the production costs. Although batch fermentation under nonsterile conditions was successful, the performance of the continuous (repeated batch) fermentation process was not ideal. Therefore, future research should focus on the combination of multiple antibacterial methods, such as appropriately designing alternative nitrogen sources or improving the ability of engineered strains to resist high osmotic pressures to further improve the antipollution performance of *C. glutamicum*, which will further increase the competitiveness of *C. glutamicum* in industrial fermentation.

## Supporting Information Available

DNA sequences of genes used in this study; l-lysine production and sugar consumption in repeated batch fermentation by CgΔexeR*Pst in the nonsterile Pt medium; and l-lysine production and sugar consumption in immobilized continuous (repetitive batch) fermentation by CgΔexeR*Pst in the nonsterile Pt medium (PDF)
