# Supplementary material for: Nonsterile l-Lysine Fermentation Using Engineered Phosphite-Grown Corynebacterium glutamicum
Source: ACS Omega. 2021 Apr 7;6(15):10160–7. doi: 10.1021/acsomega.1c00226 (PMC8153679; doi:10.1021/acsomega.1c00226)
Supplement: Supplementary file 1 — ao1c00226_si_001.pdf [file ao1c00226_si_001.pdf]

Non-sterile L-lysine fermentation using engineered phosphite-grown *Corynebacterium glutamicum*

Ming Lei[1,2], Xiwei Peng[1,2], Wenjun Sun [1,2], Di Zhang[1,2], Zhenyu Wang[1,2], Zhengjiao Yang[1,2], Chong Zhang[1,2], Bin Yu[1,2], Huanqing Niu[1,2], Hanjie Ying[1,2,3], Pingkai Ouyang[1,2], Dong Liu[1,2,3*], Yong Chen[1,2*]

[1] National Engineering Research Center for Biotechnology, College of Biotechnology and Pharmaceutical Engineering, Nanjing Tech University, Nanjing, China

[2] State Key Laboratory of Materials-Oriented Chemical Engineering, College of Biotechnology and Pharmaceutical Engineering, Nanjing Tech University, Nanjing, China

[3] School of Chemical Engineering and Energy, Zhengzhou University, Zhengzhou 450000, China

Table S1. DNA sequences of genes used in this study.

Genes	Sequence
ptxD$_{Pst}$	ATGCTGCCAAAGTTGGTGATCACCCACCGCGTCCACGACGAA<br>ATCCTGCAGTTGCTGGCACCGCACTGCGAACTTATGACCAAC<br>CAGACCGACTCCACCCTGACCCGTGAAGAAATCCTGCGCCGT<br>TGCCGTGATGCACAGGCAATGATGGCATTCATGCCAGATCGC<br>GTTGATGCAGATTTCCTGCAGGCATGCCCAGAACTGCGCGTT<br>GTTGGTTGCGCACTTAAGGGTTTCGACAACTTCGATGTCGAT<br>GCATGCACCGCCCGCGGTGTTTGGCTTACCTTCGTTCCAGATT<br>TGCTCACCGTCCCAACCGCAGAACTCGCAATTGGCCTGGCTG<br>TTGGCCTGGGTCGTCACCTTCGTGCAGCAGATGCATTCGTGC<br>GTTCCGGTGAATTCCAGGGCTGGCAGCCACAGTTCTACGGTA<br>CCGGTCTGGATAACGCTACCGTGGGTATTTTGGGTATGGGCG<br>CAATTGGCCTCGCAATGGCAGATCGTCTGCAGGGTTGGGGTG<br>CAACCCTTCAGTACCACGAAGCAAAGGCTCTGGACACCCAGA<br>CCGAGCAGCGTCTTGGTTTGCGTCAGGTGGCATGTTCCGAAC<br>TCTTCGCGTCCTCCGATTTCATCCTGCTGGCACTGCCACTCAA<br>CGCTGATACCCAGCACCTGGTGAACGCTGAATTGCTGGCACT<br>GGTTCGTCCTGGCGCATTGTTGGTGAACCCATGTCGTGGTTCT<br>GTGGTTGATGAAGCTGCTGTTTTGGCAGCACTGGAACGTGGC<br>CAGCTCGGTGGTTACGCAGCAGATGTTTTCGAAATGGAAGAT<br>TGGGCACGCGCTGATCGCCCTCGTCTTATTGATCCTGCACTGC<br>TGGCTCACCCTAACACCCTTTTCACCCCACACATCGGCTCTGC<br>AGTGCGTGCAGTTCGTCTTGAAATCGAGCGCTGTGCAGCACA<br>GAACATCATCCAGGTCCTGGCAGGCGCACGTCCAATTAACGC<br>AGCAAACCGCCTCCCTAAAGCAGAACCAGCCGCATGTTAA
ptxD$_{Pae}$	ATGCTGCCGAAACTCGTTATAACTCACCGAGTACACGATGAG<br>ATCCTGCAACTGCTGGCGCCACATTGCGAGCTGATGACCAAC<br>CAGACCGACAGCACGCTGCCGCGCGAGGAAATTCTGCGCCG

CTGCCGCGATGCTCAGGCGATGATGGCGTTCATGCCCGATCG
GGTCGATGCAGACTTTCTTCAAGCCTGCCCTGAGCTGCGTGT
AGTCGGCTGCGCGCTCAAGGGCTTCGACAATTTCGATGTGGA
CGCCTGTACTGCCCGCGGGGTCTGGCTGACCTTCGTGCCTGA
TCTGTTGACGGTCCCGACTGCCGAGCTGGCGATCGGACTGGC
GGTGGGGCTGGGGCGGCATCTGCGGGCAGCAGATGCGTTCGT
CCGCTCTGGCAAGTTCCAGGGCTGGCAACCACAGTTCTACGG
CACGGGGCTGGATAACGCTACGGTCGGCATCCTTGGCATGGG
CGCCATCGGACTGGCCATGGCTGATCGCTTGCAGGGATGGGG
CGCGACCCTGCAGTACCACGAGGCGAAGGCTCTGGATACAC
AAACCGAGCAACGGCTCGGCCTGCGCCGGGTGGCGTGCAGC
GAACTCTTCGCCAGCTCGGACTTCATCCTGCTGGCGCTTCCCT
TGAATGCCGATACCCAGCATCTGGTCAACGCCGAGCTGCTTG
CCCTCGTACGGCCGGGCGCTCTGCTTGTAAACCCCTGTCGTG
GTTCGGTAGTGGATGAAGCCGCCGTGCTCGCGGCGCTTGAGC
GAGGCCAGCTCGGCGGGTATGCGGCGGATGTATTCGAAATG
GAAGACTGGGCTCGCGCGGACCGGCCGCGGCTGATCGATCCT
GCGCTGCTCGCGCATCCGAATACGCTGTTCACTCCGCACATA
GGGTCGGCAGTGCGCGCGGTGCGCCTGGAGATTGAACGTTGT
GCAGCGCAGAACATCATCCAGGCATTGGCAGGTGCGCGCCC
AATCAACGCTGCGAACCGTCTGCCCAAGGCCGAGCCTGCCGC
ATGTTGA

ATGCTGCCAAAGTTGGTGATCACCCACCGCGTCCACGACGAA
ATCCTGCAGTTGCTGGCACCGCACTGCGAACTTGTGACCAAC
CAGACCGACTCCACCCTGACCCGTGAAGAAATCCTGCGCCGT
ptxD$_{Kpn}$ TGCCGTGATGCCCAGGCAATGATGGCATTCATGCCAGATCGC
GTGGACGCAGATTTCCTGCAGGCATGTCCAGAACTCCGCGTG
GTTGGTTGCGCACTTAAGGGTTTCGATAACTTCGATGTCGAT
GCATGCACCGCACGTGGCGTTTGGCTTACCTTCGTGCCAGAT

	TTGCTTACCGTCCCAACCGCAGAACTCGCAATTGGCTTGGCT
	GTTGGCCTGGGTCGTCACCTTCGTGCAGCAGATGCATTCGTG
	CGCTCCGGTGAATTCCAGGGTTGGCAGCCACAGTTCTACGGC
	ACCGGTCTTGATAACGCTACCGTGGGTATTCTGGGCATGGGC
	GCAATTGGCCTTGCTATGGCAGAACGTCTCCAGGGTTGGGGT
	GCAACCCTTCAGTACCACGAAGCAAAGGCACTGGACACCCA
	GACCGAACAGCGTCTTGGTCTCCGTCAGGTGGCATGTTCCGA
	ACTCTTCGCGTCCTCCGATTTCATCCTGCTGGCGCTTCCACTC
	AACGCAGATACCCAGCACTTGGTGAACGCAGAACTGCTGGC
	ACTTGTCCGCCCAGGTGCACTTCTTGTTAACCCATGCCGCGGT
	TCCGTCGTTGATGAAGCTGCTGTGCTCGCTGCCCTGGAACGT
	GGTCAGCTTGGTGGTTACGCAGCAGATGTTTTCGAAATGGAA
	GATTGGGCACGCGCAGATCGCCCACGTCTTATTGATCCAGCA
	CTGCTGGCACACCCTAACACCTTGTTCACCCCACACATCGGC
	TCCGCAGTGCGTGCAGTTCGTCTTGAAATTGAGCGCTGTGCA
	GCACAGAACATCATCCAGGTCCTGGCAGGCGCACGTCCAATT
	AACGCAGCAAACCGCCTGCCAAAAGCTGAACCAGCAGCATG
	TTAA
	AGATCAGTAGGCGCGTAGGGTAAGTGGGGTAGCGGCTTGTTA
	GATATCTTGAAATCGGCTTTCAACAGCATTGATTTCGATGTAT
	TTAGCTGGCCGTTACCCTGCGAATGTCCACAGGGTAGCTGGT
P*eftu*	AGTTTGAAAATCAACGCCGTTGCCCTTAGGATTCAGTAACTG
	GCACATTTTGTAATGCGCTAGATCTGTGTGCTCAGTCTTCCAG
	GCTGCTTATCACAGTGAAAGCAAAACCAATTCGTGGCTGCGA
	AAGTCGTAGCCACCACGAAGTCCAGGAGGACATACA

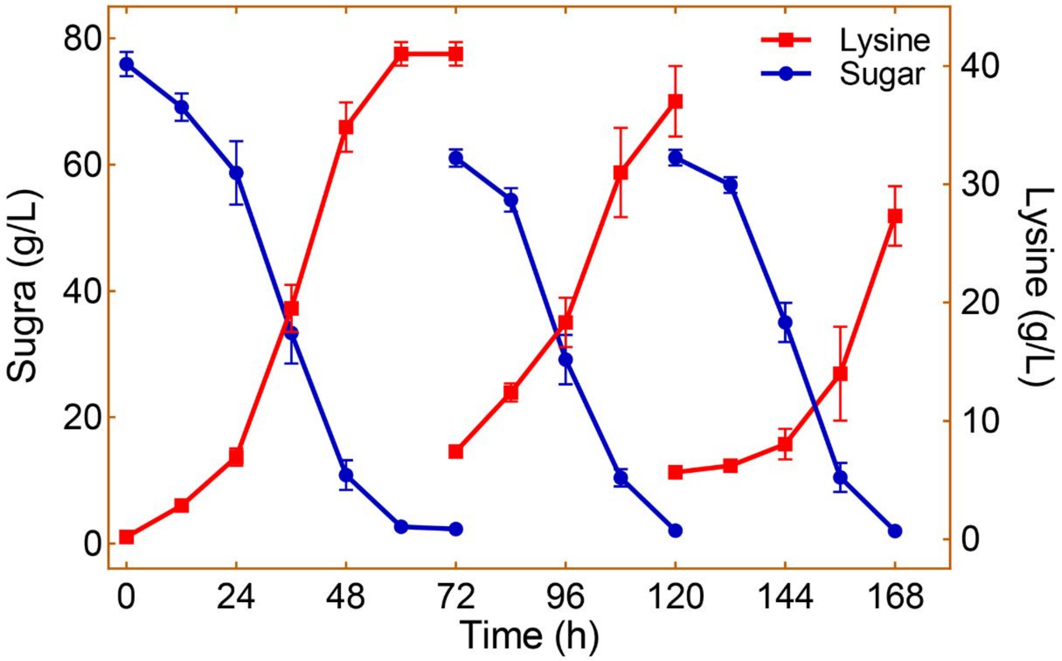

Figure S1. L-lysine production and sugar consumption in repeated batch fermentation by CgΔexeR*Pst in non-sterile Pt medium. Error bars are given showing standard deviations for n = 3.

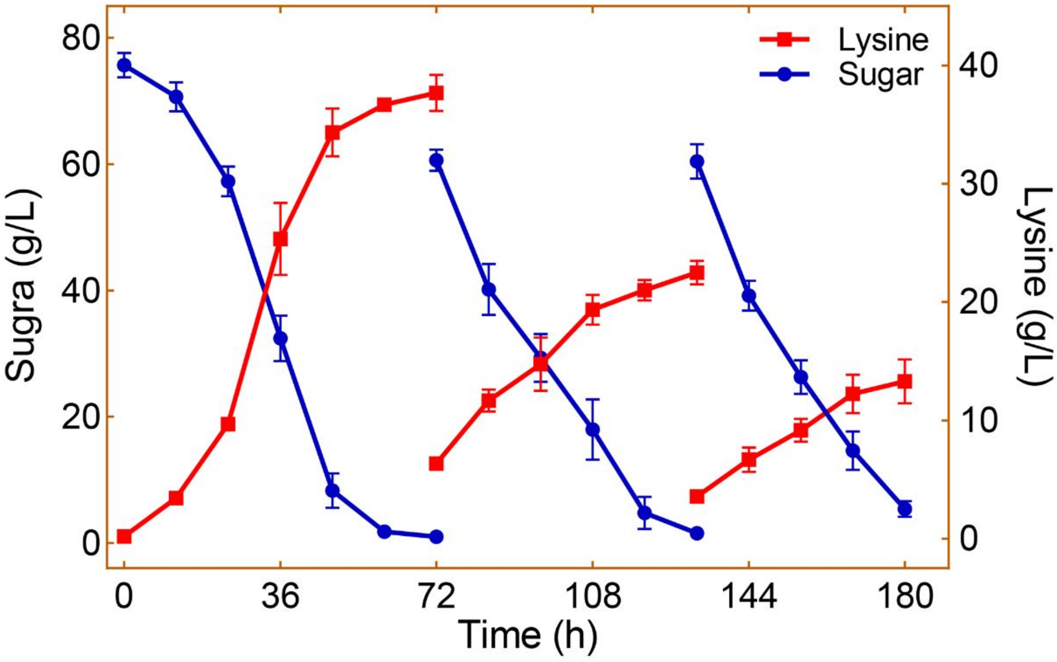

Figure S2. L-lysine production and sugar consumption in immobilized continuous (repetitive batch) fermentation by CgΔexeR*Pst in non-sterile Pt medium. Error bars are given showing standard deviations for n = 3.
